# New adenovirus-based vaccine vectors targeting Pfs25 elicit antibodies that inhibit *Plasmodium falciparum* transmission

**DOI:** 10.1186/s12936-017-1896-7

**Published:** 2017-06-15

**Authors:** Kathleen A. McGuire, Kazutoyo Miura, Christopher M. Wiethoff, Kim C. Williamson

**Affiliations:** 1 Department of Microbiology and Immunology, Stritch School of Medicine, Loyola University Chicago, Maywood, IL 60153 USA; 2 Laboratory of Malaria and Vector Research, National Institute of Allergy and Infectious Diseases, National Institutes of Health, Rockville, MD USA; 3 Department of Microbiology and Immunology, Uniformed Services University, Bethesda, MD 20814 USA; 4 Abbvie, 1 North Waukegan Road, North Chicago, IL 60064 USA; 5 Eli Lilly and Company, Lilly Corporate Center, Indianapolis, IN 46285 USA

**Keywords:** Malaria, *P. falciparum*, Transmission-blocking immunity, Pfs25, Adenovirus vaccine vectors

## Abstract

**Background:**

An effective malaria transmission-blocking vaccine (TBV) would be a major advance in the current efforts to eliminate and, ultimately, eradicate malaria. Antibodies against *Plasmodium falciparum* surface protein, Pfs25, are known to block parasite development in the mosquito vector. However, in initial clinical trials the limited immunogenicity of recombinant Pfs25 protein-in-adjuvant vaccines has been a challenge.

**Methods:**

Novel human adenovirus type 5 (Ad5) vectors were used in heterologous prime boost vaccination strategies to augment the immune response against Pfs25. Specifically, an Ad5 vector that directs expression of full-length, membrane-bound Pfs25 was used as a priming immunization followed by a boost with Ad5 viral particles displaying only the Pfs25 epitope targeted by transmission-blocking antibodies 4B7 and 1D2 (Pfs25 aa 122–134) in hypervariable region 5 of the hexon capsid protein.

**Results:**

This heterologous prime-boost vaccine strategy induced antibodies that significantly inhibit *P. falciparum* transmission to mosquitoes in a standard membrane-feeding assay. Further, immunized mice generated a robust anti-Pfs25 antibody response characterized by higher titer, higher relative avidity and a broader IgG subclass profile than observed with a homologous prime-boost with recombinant Pfs25/alum.

**Conclusion:**

The data suggest that focusing the immune response against defined epitopes displayed on the viral capsid is an effective strategy for transmission-blocking vaccine development.

## Background

The *Plasmodium* parasites that cause malaria continue to be a risk for 40% of the world’s population despite the introduction of artemisinin-based combination therapy and enhanced vector control measures a decade ago [1]. In addition to the approximately 200 million clinical cases a year, many infections in endemic countries are asymptomatic. Both symptomatic and asymptomatic infections produce sexual stage parasites, called gametocytes, required for malaria transmission [2–4]. This large infectious reservoir coupled with the lack of efficacy of common anti-malarials against gametocytes highlights the need for new approaches to block the spread of the disease [5]. Mass drug administration campaigns have been shown to decrease malaria transmission in isolated areas, but are challenging to implement and even more difficult to sustain [6, 7]. The development of a vaccine that effectively blocks malaria transmission would enhance control strategies and also provide protection against the spread of drug- or vaccine-resistant lines.

Pfs25 is currently the most advanced transmission-blocking vaccine (TBV) candidate [8, 9]. This 25 kD surface protein is expressed after the gametocyte is taken up in a blood meal by a mosquito and stimulated to emerge as a gamete [10–12]. Pfs25 expression continues through fertilization and differentiation into an ookinete and is thought to aid in ookinete migration across the mosquito midgut to form an oocyst [13, 14]. Pfs25-specific monoclonal antibodies (mAb) with potent transmission-blocking activity have been identified and the epitopes of two mAbs have been mapped to Pfs25^121–130^ (ILDTSNPVKT), but this defined peptide has not been tested directly as a vaccine candidate [15–17]. To date, the recombinant protein-based vaccine candidates tested in humans show low immunogenicity; therefore, new approaches are needed to generate high titer antibody responses targeting transmission-blocking epitopes [8, 9]. In this study, Ad5-based vaccine vectors were developed to direct the Pfs25-specific antibody response to relevant, transmission-blocking epitopes.

Replication-defective adenoviruses (Ad) are an attractive vaccine platform due to their ability to potently activate the immune system [18, 19]. In addition to their immuno stimulatory properties, Ad vectors have well-characterized physical properties, a relatively stable viral capsid, a genetically tractable genome that does not integrate, and can be propagated to high titer in vitro. Specifically, Ad vectors can express exogenous antigens from a transgene or can display antigens within the viral capsid itself [19, 20]. A heterologous prime boost strategy using an Ad-*pfs25* virus followed by a modified vaccinia Ankara (MVA)-*pfs25* virus that both direct secretion of the full length Pfs25 exodomain has been shown to induce antibodies that inhibit *Plasmodium falciparum* transmission [21]. Recently, in an effort to enhance Ad-*pfs25/*MVA-*pfs25* immunogenicity, the IMX313 peptide coding sequence was added to the C-terminus of the Pfs25 gene in both viral vectors resulting in the secretion of a self-assembling heptamers [22].

In this study, novel Ad5-based TBVs targeting Pfs25 were generated that induce antibodies in mice that significantly inhibit transmission of *P. falciparum* to *Anopheles* mosquitoes in a standard membrane-feeding assay (SMFA). Specifically, an Ad5 vector directing expression of full-length, membrane-bound Pfs25 used as a priming immunization was found to generate a high titer, high relative avidity Pfs25 antibody response with broad IgG subclasses. A boost immunization with an Ad5-vector displaying Pfs25 transmission-blocking epitope D3 (Pfs25 aa 122–134) within the viral capsid further increased Pfs25-specific antibody titer, relative avidity, and transmission-blocking activity when compared to homologous prime-boost with alum-adjuvanted Pfs25. This approach demonstrates the efficacy of expressing a discrete epitope within the viral capsid and provides a new strategy to enhance transmission-blocking immunity.

## Methods

### Cell lines and culture

HeLa cells were obtained from ATCC (Manassas, VA, USA), 293β5 cells, stably expressing β5 integrin, were a gift from Glen Nemerow, and 293 T-REx cells were purchased from Life Technologies (Carlsbad, CA, USA). Tissue culture reagents were obtained from Mediatech (Manassas, VA, USA) and HyClone (Erie, UK). HeLa cells, 293β5 cells and 293 T-REx cells were maintained in Dulbecco’s Modified Eagle Medium (DMEM) supplemented with 1 mg/ml streptomycin, 100 IU/ml penicillin, 0.25 mg/ml amphotericin B, non-essential amino acids, 2 mM glutamine, 10 mM Hepes buffer, 1 mM sodium pyruvate. 293Trex cells were maintained with 5 μg/ml blasticidin (Sigma Aldrich, St Louis, MO, USA). Unless specified all other reagents were from Thermo Fisher Scientific (Waltham, MA, USA).

### Virus generation

Ad5gfp generation was reported before [23] and the Ad5-*pfs25* virus was generated using a modified AdEasy system as described previously [24, 25]. Briefly, the Pfs25 gene (bp 1–217) was codon-optimized for expression in humans (Genscript, Piscataway, NJ, USA) and inserted it into the pshuttle-CMV vector (Agilent Technologies plasmid #240007, Santa Clara, CA, USA). Lambda red recombineering was used to insert the pshuttle-CMV vector containing the Pfs25 gene into the E1/E3-deleted Ad5 genome. After finding that Pfs25 expression decreased viral yield by at least tenfold, the tet operator (TO) sequence from pcDNA4/TO/myc-HisA was inserted upstream of the cytomegalovirus (CMV) promoter. Inserting TO allowed suppression of Pfs25 expression in 293Trex cells during propagation and increased viral yield. For the Ad5-HVR-*pfs25* vectors, galk recombineering with positive and negative selection steps was used as previously described [26]. Briefly, galk was amplified from the pgalk plasmid (obtained from the NCI BRB Preclinical Repository, Frederick, MD, USA) using primers containing homology to regions within the hexon capsid protein hypervariable regions 1 (HVR1) or 5 (HVR5) flanking the galk sequence to replace amino acids 145–158 and 266–278, of HVR1 or HVR5, respectively, into the in the Ad5 genome (Table 1).

**Table 1:** Primer sequences for recombineering

Primer name	Sequence
Primers for galk insertion	
HVR1 galk forward	gtgccccaaatccttgcgaatgggatgaagctgctactgctcttgaaatacctgttgacaattaatcatcggca
HVR1 galk reverse	agaataaggcgcctgcccaaatacgtgagttttttgctgctcagcttgcttcagcactgtcctgctcctt
HVR5 galk forward	agcaacaaaatggaaagctagaaagtcaagtggaaatgcaatttttctcacctgttgacaattaatcatcggca
HVR5 galk reverse	gtgtctggggtttctatatctacatcttcactgtacaataccactttaggtcagcactgtcctgctcctt
Primers for pfs25 epitope insertion	
HVR1 DIII forward	gtgccccaaatccttgcgaatgggatgaagctgctactgctcttgaaataatctggatacatctaatcccgtgaagactggagtctgcagt
HVR1 DIII reverse	agaataaggcgcctgcccaaatacgtgagttttttgctgctcagcttgctcacaactgcagactccagtcttcacgggattagatgtatc
HVR5 pfsDII forward	agcaacaaaatggaaagctagaaagtcaagtggaaatgcaatttttctcaattgatgggaacccagtgtcctacgcctgcaagtgtaat
HVR5 pfsDII reverse	gtgtctggggtttctatatctacatcttcactgtacaataccactttaggattacacttgcaggcgtaggacactgggttcccatcaat
HVR5 pfsDIII forward	agcaacaaaatggaaagctagaaagtcaagtggaaatgcaatttttctcactggatacatctaatcccgtgaagactggagtctgcagt
HVR5 pfsDIII reverse	Gtgtctggggtttctatatctacatcttcactgtacaataccactttaggacaactgcagactccagtcttcacgggattagatgtatc

Using a second round of recombineering, galk was replaced with a peptide epitope from Pfs25 epidermal growth factor (EGF) domain II, Pfs25^83–95^ (referred to as D2); or domain III, Pfs25^122–134^ (referred to as D3) using primer dimers of complementary oligonucleotides encoding the entire peptide sequence (flanked by sequences homologous to either HVR1 or HVR5 (Table 1). Positive recombinants were confirmed by restriction enzyme digest, PCR amplification of the *pfs25* epitopes and sequencing of the Hexon region of the genome. The same number of amino acids were removed from the Ad5 Hexon as were inserted to conserve the size of the HVR domains.

### Virus preparation

With the exception of Ad5-*pfs25* (propagated in 293Trex cells) all viruses were propagated in HEK293 cells, then purified by two rounds of cesium chloride gradient centrifugation twice and dialyzed in 40 mM Tris, 150 mM NaCl, 10% glycerol, and 1 mM MgCl_2_ (pH 8.2) [27]. Viral concentration was determined by Bradford assay using 1 mg of protein per 4 × 10^9^ viral particles as the conversion factor (Bio-Rad Laboratories, Inc., Hercules, CA, USA). To determine viral titer, the viruses were serially diluted on HeLa cells and green fluorescent protein (GFP) expression quantified by flow cytometry. For the Ad5-*pfs25* lacking the GFP reporter, the titer was determined by immunofluorescence assay (IFA) detecting adenovirus Hexon in 293β5 cells with a dylight labelled 9C12 antibody (Developmental Studies Hybridoma Bank, University of Iowa, DSHB Product TC31-9C12.C9, Iowa City, IA, USA). For the viruses used in this study, specific infectivity ranged from 100 to 200 viral particles per GFP-transducing unit.

### Pfs25 in Ad5 vectors

To confirm Pfs25 expression from Ad5-*pfs25*, HeLa cells were transfected on glass cover slips in a 24-well plate (100,000 cells per well) and 24 h later fixed with 3.7% paraformaldehyde—0.159 M PIPES [piperazine-*N*,*N*′-bis(2-ethanesulfonic acid)] buffer (Sigma) for 15 min, blocked in phosphate buffered saline (PBS) with 10% fetal bovine serum (FBS) and 0.5% saponin (Sigma), permeabilized with 0.5% Triton X-100 (Sigma), and washed with PBS. After blocking for 1 h in 10% FBS, the cells were probed with ID2, a monoclonal antibody recognizing a conformational epitope in EGF domain III of Pfs25 [28], followed by an Alexa fluor 488-conjugated anti-mouse IgG antibody (Invitrogen, Carlsbad, CA, USA).

### Mice and immunizations

All studies were reviewed and approved by the Institutional Animal Care and Use Committee of Loyola University Chicago (Maywood, IL) under IACUC protocol number 2011020. 6–12 week old male C57/BL6 wild type mice (Jackson laboratories, Bar Harbor, ME) were vaccinated intramuscularly in the left quadriceps and serum was collected 10 or 21 days following the last immunization. For protein immunizations, purified Pfs25 was adsorbed to aluminum hydroxide (Alhydrogel from Invivogen, San Diego, CA, USA) at a 1:1 ratio (in weight) of protein to alum in solution. In the first animal study, mice were immunized with 2.5 μg of yPfs25 or 10^10^ viral particles of Ad5-*pfs25* on day 0 and sera and splenocytes were collected on day 10. In the second study, mice were immunized with 25 μg of yPfs25 (to increase detectable antibody titer) or 10^10^ viral particles of Ad5-*pfs25*, Ad5-HVR5D2, or Ad5-HVR5D3 on day 0 and sera were collected on day 21. The third study was a prime-boost vaccination study, where mice were immunized on days 0 and 21 and serum samples were collected 21 days after the boost. In the prime-boost study, protein immunizations contained 2.5 μg Pfs25-alum, Ad5-*pfs25* was administered at 10^9^ viral particles, and all Ad5-HVR-*pfs25* vaccinations were administered at 10^10^ viral particles per animal.

### Determination of Pfs25-specific serum antibody titer and IgG subclass production

Blood was collected via cardiac puncture and the serum was isolated, aliquoted, and stored at −80 °C until use. Serum from each vaccination group were pooled to allow direct comparison between the enzyme-linked immunosorbent assay (ELISA)s and later SMFA. Individual ELISAs compared each vaccine group in triplicate. High-binding ELISA plates (Costar cat # 07-200-35, Sigma) were coated with Pfs25 protein at 1 μg/ml in coating buffer (eBioscience, San Diego, CA, USA) overnight at 4 °C. The plates were then blocked with 1× assay diluent (AD) (eBioscience 00-4202-56) for 1 h, washed with PBST (0.05% Tween), and added serially diluted serum (in 1× AD) for 2 h followed by anti-mouse IgG (Fc) (Abcam, ab97265, Cambridge, MA, USA) at 1:5000 for 1 h. 1xTMB substrate (eBioscience cat # 00-4201-56) was added and the reaction was stopped with 1 M sulfuric acid, before measuring the colourimetric change at 450 nm. Antibody endpoint titers (depicted as ELISA units) were determined using an absorbance value 3 standard deviations above the PBS-immunized control mice. For the IgG subclass ELISAs all steps were performed as described above except serum samples were added at one constant dilution (1:500) and the secondary antibodies (1:500 dilution) added were specific to IgG1, IgG2a or IgG3 (Southern Biotech, cat # 5300-05B, Birmingham, AL, USA).

### Determining the relative avidity of Pfs25-specific serum antibodies

The avidity assay is a modified Pfs25-specific ELISA where plates were coated with yPfs25 and serum was added to the wells. In this case, the plates were incubated with a fixed dilution of serum for 2 h at a dilution where the antibody binding reaches saturation (determined previously by assessing the O.D. for various dilutions from the antibody titer ELISA). Following serum incubation and washes, increasing concentrations of NaSCN were added starting from 0.25 to 4 M diluted in PBS (Sigma) and incubated at room temperature for 15 min with shaking. The plate was then washed 15 times with PBST before adding the secondary antibody [anti-mouse IgG (Fc)] and detection as previously described. For each sample, the NaSCN concentration (IC_50_) at which the O.D. value was reduced by 50% compared to the O.D. when no NaSCN was added was calculated.

### Ex vivo splenocyte stimulation and cytokine production

Splenocytes were harvested from mice immunized with Ad5-*pfs25* or yPfs25-alum 10 days post-immunization (the first animal study). Following red blood cell lysis, cells were added to a 96-well tissue culture plate (Costar, Sigma) with specific antigens in RPMI. Whole splenocytes were restimulated with Ad5 (10,000 particles per cell), yPfs25 (10 μg/ml), or Concanavalin A (ConA, 2.5 μg/ml) as a positive control or no antigen as the unstimulated control. After 24 h, cell-free supernatants were collected and subjected to an ELISA to detect IFNγ secretion following theeBioScience IFNγ ready-set-go ELISA protocol.

### Generation of the Pfs25 homology model

The homology modeling software, SWISS-MODEL was used to generate a model of the Pfs25 extracellular domain [29]. The primary sequence of Pfs25^23–217^ was threaded onto the structure of Pvs25 [10] and further optimized through energy minimization algorithms selected by the software. Pvs25 is ~46% identical to Pfs25 within this sequence. Disulfide bonding patterns present in the crystal structure of Pvs25 were maintained by the Pfs25 model.

### Standard membrane-feeding assays (SMFAs)

The standard membrane-feeding assay (SMFA) to assess transmission blockade has been previously described [30]. In brief, *P. falciparum* (NF54 strain) was cultured for 16–18 days to produce mature gametocytes in vitro. Mature gametocytes (adjusted to ~0.15% of Stage V gametocytaemia) were mixed with test serum diluted 1:10 with PBS, and fed to ~50 female *Anopheles stephensi.* Mosquitoes were kept for 8 days, and dissected (n = 20 per sample) to enumerate oocysts in their midguts. Only midguts from mosquitoes with any eggs at the time of dissection were analyzed. Percent inhibition in oocyst intensity was calculated as: 100 × {1 − (mean number of oocysts in the test)/(mean number of oocysts in the control)}.

### Statistics

Splenocyte IFNɣ production was compared between two groups using a Student’s T test. For the SMFA, the best estimates of percent inhibition, the 95% confidence interval and the p-value of each test sample from single or multiple feeds were calculated based on the model generated in the previous study [30]. Using the same model, ratios of oocyst numbers among test groups were calculated.

## Results

### Vaccination with Ad5 expressing Pfs25 generates high titer anti-Pfs25 antibodies consistent with T cell help

**Fig. 1:**
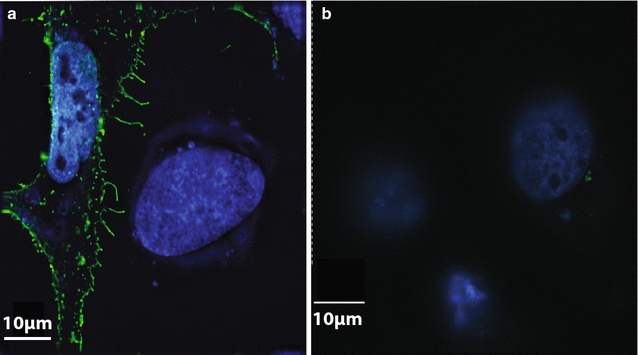
Pfs25 expression on Hela cells. Hela cells were transfected with an Ad5 shuttle vector with (**a**) or without (**b**) the codon-optimized Pfs25 gene under the control of the CMV immediate early promotor. Twenty-four hours post transfection, cells were fixed, permeabilized, and probed with ID2, a conformation-dependent Pfs25-specific monoclonal antibody, then probed with an Alexa Fluor488-conjugated anti-mouse secondary antibody and visualized by IFA

**Fig. 2:**
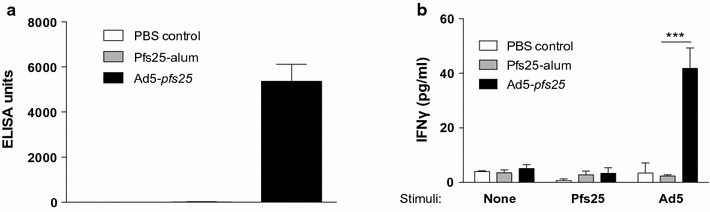
Pfs25 specific Ig production and T-cell activation following Ad5-*pfs25* primary vaccination. Mice (n = 3 per group) were immunized with Ad5 expressing Pfs25 (10^10^ particles) or recombinant yPfs25-alum (2.5 µg/mouse) on day 0, and serum and splenocyte samples were collected on day 10. **a** yPfs25-specific ELISA, yPfs25-specific ELISA were performed and data depict pooled serum from 3 animals per group tested in triplicate in 3 independent ELISAs. The average ELISA unit and standard deviation are plotted. **b** Splenocyte IFNγ secretion. Splenocytes (n = 3, tested individually) were incubated without or with yPfs25 at 10 μg/ml or Ad5 at 10,000 particles per cell for 24 h as indicated on the *x*-*axis*. IFNγ secretion was detected by ELISA and the average and standard deviation are plotted. Statistical significance was determined by Student’s T test; ***p < 0.001

In this study, several novel Ad5-based transmission blocking vaccine vectors were generated in an effort to stimulate a high titer antibody response against Pfs25. An Ad5 vector encoding full length Pfs25 (aa 1–217) under the CMV promoter was generated first. This Pfs25 construct includes the proposed endogenous Pfs25 secretory signal (aa 1–21) and glycosyl-phosphatidylinositol (GPI)-anchor addition signal (aa 194–217), which directs trafficking to and retention on the plasma membrane and consequently may enhance B cell activation. After confirming the Ad5-*pfs25* vector sequence, HeLa cells were transfected with the Ad5-*pfs25* genome and 24 h post-transfection the majority of Pfs25 concentrated at the periphery of the cells using Pfs25-specific, transmission-blocking monoclonal ID2 suggestive of cell surface localization [28] (Fig. 1). Mice were immunized with Ad5-*pfs25* (10^10^ viral particles) or a current transmission blocking vaccine candidate, yPfs25 (2.5 μg) adsorbed to aluminum hydroxide (yPfs25-alum) and the Pfs25 immune response was compared. yPfs25-specific antibodies were detected 10 days post-immunization with Ad5-*pfs25* (Fig. 2a). Splenocytes harvested 10-days post Ad5-*pfs25* vaccination exhibited a significant increase in adenovirus-specific IFNγ secretion following restimulation with Ad5-*pfs25* but not following yPfs25 stimulation (Fig. 2b).These data suggest Ad5-*pfs25*-immunized mice generated Ad-specific T cells, which may help B cell responses to Pfs25 (Fig. 2b). In contrast, splenocytes from yPfs25-alum-immunized mice did not exhibit IFNγ production upon Ad5-*pfs25* stimulation ex vivo (Fig. 2b) and neither immunization elicited detectable Pfs25-specific T cell responses as assessed by monitoring IFNɣ (Fig. 2b).

### Primary Ad5-*pfs25* vaccination generates a high avidity Ig response with a broad IgG subclass profile

**Fig. 3:**
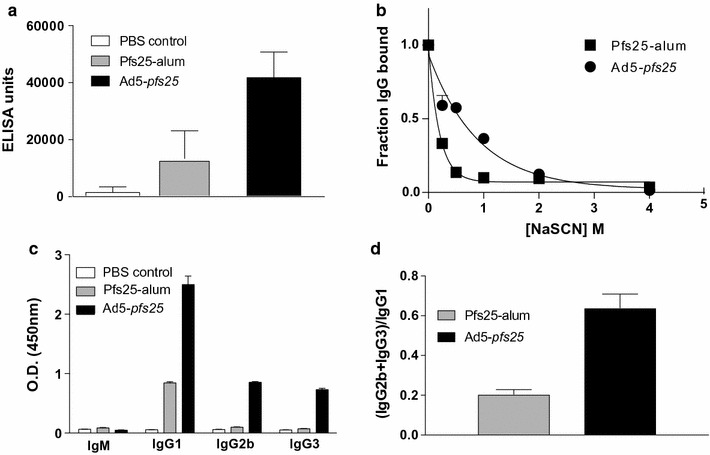
Pfs25-specific serum antibody response after primary immunization with an Ad vector expressing Pfs25. Mice (n = 3 per group) were immunized with Ad5 expressing Pfs25 (10^10^ particles) or recombinant yPfs25-alum (25 μg/mouse) on day 0, and serum samples were collected on day 21. **a** yPfs25-specific antibody titer. yPfs25-specific ELISA was performed and data depict pooled serum from 3 animals per group tested in triplicate in 3 independent ELISAs. The average ELISA unit and standard deviation are plotted. **b** yPfs25-specific avidity. Sera were added to a yPfs25-coated ELISA plate at a single dilution and then incubated with increasing concentrations of NaSCN. The remaining yPfs25-specific antibodies were detected with an HRP-conjugated anti-mouse IgG (Fc). The fraction of Ig bound was determined by dividing the optical density (OD) at each NaSCN concentration by the OD in the absence of NaSCN and the nonlinear best-fit line was plotted for each data set. **c**, **d** yPfs25-specific IgM and IgG subclass profile. yPfs25-specific ELISA was performed and the yPfs25-specific IgM and IgG subclasses were detected with HRP-conjugated specific antibodies. The average OD and standard deviation for each isotype/subclass (**c**) and the ratio of the O.D. for the IgG1 to the O.D.s of IgG2b and IgG3 (**d**) are shown

To further characterize the Pfs25-specific antibody response, a second immunization study was conducted and assessed the relative avidity and IgG subtype profile following vaccination at the peak of the antibody response 21 days post-immunization. In this regimen the dose of yPfs25 protein was increased tenfold to 25 μg to generate a detectable Pfs25-specific antibody response to compare with Ad5-*pfs25* immunization. Under these conditions 21 days post-immunization, both vaccinations elicit anti-yPfs25 antibody titers (Fig. 3a). Antibodies elicited by Ad5-*pfs25* had a higher relative avidity to yPfs25 than those induced by yPfs25/alum vaccination as demonstrated by their fourfold greater IC_50_ (Fig. 3b). In addition to higher relative avidity, Ad5-*pfs25* vaccination generated a broader array of IgG subclasses (Fig. 3c, d), while yPfs25-alum primarily induced anti-yPfs25 IgG1 titers, which is not unexpected from an alum-adjuvanted protein vaccine.

### Heterologous prime-boost vaccination enhances the Pfs25-specific antibody response

**Fig. 4:**
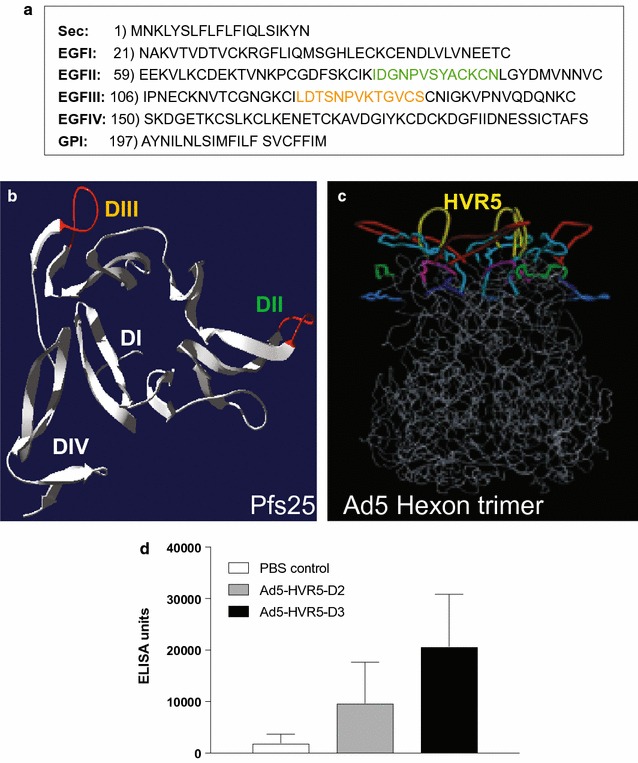
Pfs25 epitope specific vectors design. **a** Amino acid sequence of Pfs25. The selected Pfs25 EGF domains II and III from which the epitope peptides (denoted as D2 and D3) were chosen for HVR insertion are indicated in *green* and *orange*, respectively. **b** Predicted Pfs25 structure based on the structure of the *P. vivax* homolog, Pvs25. The D2 and D3 epitopes in EGF domains II and III, respectively, are highlighted in *red*. **c** Ad5 Hexon capsid protein structure. Ad5 Hexon trimer structure (Protein Data Bank 1P30) with the modelled HVRs highlighted (HVR1, *red*; HVR2, *green*; HVR3, *pink*; HVR4, *light blue*; HVR5, *yellow*; HVR6, *blue*; HVR7, *cyan*). **d** yPfs25-specific antibody titer. Mice (n = 3 per group) were immunized with 10^10^ viral particles of Ad5 displaying the D2 or D3 Pfs25 peptide epitope within HVR5 (denoted Ad5-HVR5D2 or Ad5-HVR5D3, respectively) or PBS control and yPfs25-specific ELISA was performed and data depict endpoint dilutions for 3 individual animals where *error bars* depict standard deviation

To examine the effect of a prime-boost vaccination strategy, a third immunization study was performed. Ad5-*pfs25* was used as the priming immunization followed by either yPfs25 or 2 novel Ad5-vectors expressing putative Pfs25 transmission-blocking epitope D2 (Pfs25^83–95^) or D3 (Pfs25^122–134^) designed to focus the immune response toward these regions within EGF-like domains II and III of Pfs25. A group of mice were immunized with yPfs25-alum (homologous prime-boost) as a comparator group. Peptide D2 is located in EGF domain II which was found to be the target of transmission-blocking antibodies induced in rabbits after yPfs25 immunization, while domain D3 is located in EGF domain III and corresponds to the epitope for the Pfs25-specific transmission-blocking mAb, 4B7 (Fig. 4b) [15–17]. Based on the crystal structure of the *Plasmodium vivax* Pfs25 paralog, Pvs25, both domains D2 and D3 are located on exposed loops in the EGF domains (Fig. 4b) [10]. Ad5 constructs were designed to display either D2 or D3 in hypervariable regions (HVR) 5 or 1 of the hexon capsid protein (Fig. 4c) resulting in the production of a virion displaying theoretically 720 copies of the inserted Pfs25 domain on its surface. Several studies demonstrate that HVR-modified virions maintain proper viral assembly, structure and infectivity and elicit a response to the antigen insertion [31, 32]. While insertion of Pfs25 epitopes into either HVR did not affect virus assembly, as determined during purification of the viral vectors, insertion into HVR1 decreased vector specific infectivity tenfold and therefore are not included in further analysis. In contrast, insertion into HVR5 had no negative effects on infectivity. A single immunization with either Ad5-HVR5D2 or Ad5-HVR5D3 induced antibodies that recognized yPfs25 recombinant protein in an ELISA assay indicating that the Pfs25 D2 or D3 epitopes were displayed on the virion and immunogenic (Fig. 4d).

**Fig. 5:**
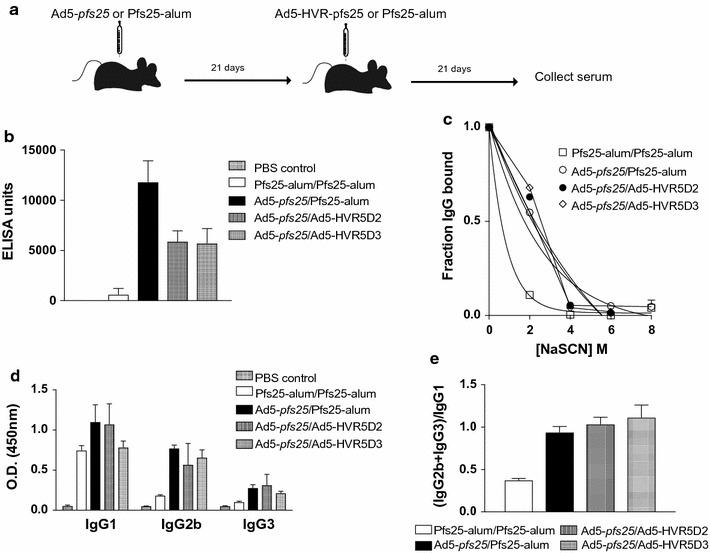
Pfs25-specific serum antibody response upon heterologous prime-boost vaccination. **a** Vaccination schematic. Mice (n = 6 per group) were immunized with yPfs25-alum (2.5 μg/mouse) or Ad5 vector Pfs25 (10^9^ particles) on day 0, and followed by boost immunization with yPfs25-alum (2.5 μg/mouse) or the different Ad5-HVR-*pfs25* vectors (10^10^ particles). The serum samples were collected 21 days after the boost. **b** yPfs25-specific antibody titer. yPfs25-specific ELISA was performed and data depict pooled serum from 6 animals per group tested in triplicate in 3 independent ELISAs. The average ELISA unit and standard deviation are plotted. **c** yPfs25-specific antibody avidity. The assay was performed as described for Fig. 3. The fraction of Ig bound was determined by dividing the OD at each NaSCN concentration by the OD in the absence of NaSCN and the nonlinear best-fit line was plotted for each data set. **d**, **e** yPfs25-specific IgG sublass profile. The assay was performed as described for Fig. 3. The average OD and standard deviation for each isotype/subclass (**d**) and the ratio of the O.D. for the IgG1 to the O.D.s of IgG2b and IgG3 (**e**) are shown. Data depict the ratio of the OD for IgG1 to the ODs of IgG2b and IgG3

In mice primed with Ad5-*pfs25*, a secondary immunization with Ad5-HVR5D2, Ad5-HVR5D3, or yPfs25-alum generated higher anti-yPfs25 IgG ELISA titers as compared to our benchmark, yPfs25-alum homologous prime-boost (Fig. 5a, b). Sera obtained after the heterologous prime-boosts also had greater relative avidity than serum obtained after homologous prime-boost with yPfs25-alum (Fig. 5c) or a single Ad5-*pfs25* immunization (Fig. 3b) as indicated by a nearly threefold higher IC_50_. Upon examining IgG subclass following prime-boost immunizations the homologous prime-boost with yPfs25-alum was found to generate an IgG1-dominated response (Fig. 5d, e) as reported previously [22, 33]. Of note, the Ad5-*pfs*25 prime and yPfs25 boost group showed similar avidity and IgG subclass profiles as the Ad5-*pfs*25 prime and Ad5-HVRD2 or D3 boost groups, rather than the yPfs25-alum homologous prime-boost group (Fig. 5c). The results suggest the Ad5-*pfs25* primary immunization determined the responses.

### Heterologous prime-boost vaccination with Ad5-based vectors blocks *P. falciparum* transmission to the mosquito vector

**Fig. 6:**
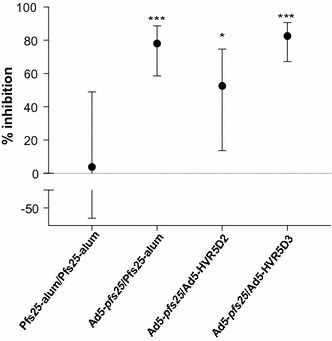
Vaccination induced transmission-blocking activity. Two independent sets of mice were immunized with the test groups, yPfs25 homologous prime boost (yPfs25-alum/yPfs25-alum) or the 3 heterologous prime boost strategies (Ad5-*pfs25*/yPfs25-alum, Ad5-*pfs25*/Ad5-HVR5D2, and Ad5-*pfs25*/Ad5-HVR5D3) as described in "Methods". Serum was pooled from the test groups from each immunization (n = 3 mice for all the immunization groups except the 2nd yPfs25 homologous prime boost immunization, which included 6 mice) and tested in SMFAs at a 1:10 dilution. Pooled serum from mice immunized with an Ad5-luciferase vector was the negative control in each assay. The first SMFA tested pooled serum from the first immunization, while the second and third SMFAs tested pooled serum from the second immunization. All three SMFAs were included in the statistical analysis. The best estimate of the percent inhibition from 3 independent SMFAs is plotted with the 95% confidence interval for each sample calculated using a zero-inflated negative binomial model [30] (***p < 0.001, *p < 0.05 as compared to baseline)

Although the heterologous prime boost vaccines enhanced the yPfs25 immune response, the critical question remaining is whether transmission-blocking efficacy increases. Therefore, transmission-blocking activity was tested by SMFA using *An. stephensi* mosquitoes [30]. Consistent with the enhanced humoral response, all the Ad5-*pfs25* prime-heterologous boost vaccines tested induced serum (tested at 1:10 dilution) that significantly reduced parasite load compared to the yPfs25-alum-immunized sera (Fig. 6). Serum harvested 21 days after the Ad5-*pfs25* prime Ad5-HVR5D3 or yPfs25-alum boost significantly reduced the average oocyst number per mosquito midgut by 82.5% (the best estimate from 3 independent SMFAs, and the 95% confidence interval (95% CI) was 67.3–90.7, p < 0.001) and 78.1% (95% CI 58.6–88.7; p < 0.001), respectively. Serum from the Ad5-*pfs25* prime Ad5-HVR5D2 boost also significantly reduced transmission, but the inhibition level was lower (52.6% inhibition; 95% CI 13.7–74.9; p = 0.023) and more variable between SMFAs. In contrast, homologous prime-boost with the same dose of yPfs25-alum did not significantly reduce transmission (3.8% inhibition; 95% CI −67.5 to 48.9; p = 0.921). The differences in oocyst numbers between homologous prime-boost with yPfs25-alum and all the Ad5-*pfs25* prime-heterologous boost vaccines were significant (p < 0.024).

## Discussion

Transmission-blocking vaccines employing Pfs25 show promising preclinical results, although effective vaccine strategies in humans remain a work in progress. In this study, new Ad5 virus-*pfs25* vectors were developed in an effort to enhance the induction of antiserum that significantly reduces malaria transmission in a SMFA. Specifically, an initial priming immunization with an Ad5-*pfs25* vector that directs expression of membrane-bound Pfs25 followed by a booster injection of Ad5 virions displaying 720 copies of a single, defined Pfs25 transmission-blocking epitope (D3) in the HVR5 of the hexon capsid protein induce antibodies that significantly inhibit *P. falciparum* oocyst production. Recombinant yPfs25 could also effectively boost the initial Ad5-*pfs25* immunization and generate antibodies that significantly reduce transmission in an SMFA. In marked contrast, serum obtained after homologous prime/boost immunizations using the same dose of recombinant yPfs25 in alum did not effectively reduce oocyst production. This differential response against yPfs25 following a homologous yPfs25 or heterologous Ad5-*pfs25* immunization demonstrates the key role of Ad5-*pfs25* in priming the immune response for the production of high avidity, high titer anti-Pfs25 antibodies. This enhanced immune response is likely due to the ability of Ad5-*pfs25* to stimulate a T cell response, which was not observed following immunization with recombinant yPfs25 in alum [34]. Previously, investigators have shown that a prime boost strategy using 2 distinct viral vectors, an adenovirus isolated from chimpanzees (ChAd63) followed by a modified Vaccinia virus (MVA), both directing expression of secreted, monomeric Pfs25, also induced transmission reducing antibodies [21]. However, this vaccination strategy generated relatively modest anti-Pfs25 antibody titers prompting the additional development of other Pfs25-targeting vaccines including Pfs25-IMX313. Pfs25-IMX313 is a secreted chimeric protein made up of Pfs25 fused to the self-associating oligomerization domain of the chicken complement inhibitor C4b-binding protein [22]. The addition of IMX313 Pfs25 to both ChAD63 and MVA Pfs25^22–193^ expression vectors enhanced immunogenicity and also improved the ability of the antiserum to block oocyst production as compared to ChAD/MVA-*pfs25* vectors without IMX313 or recombinant Pfs25-IMX313 protein. These results suggest roles for both multimerization and the viral vector in enhancing the antibody response.

This work extends the current Ad-based vector immunization strategies to evaluate Ad viral vectors that direct membrane expression of Pfs25 and focus the immune response to proposed transmission-blocking epitopes in Pfs25. To target Pfs25 expression to the surface of the cell, the Ad5-*pfs25* vector, which includes the full-length Pfs25 gene containing the both the secretory and GPI anchor signal sequence of Pfs25, was developed. These changes to the Ad-based Pfs25 expression vector resulted in multiple copies of Pfs25 expressed on the surface of the virally transduced cell [35]. Expression of membrane-bound Pfs25 is likely to improve antigen persistence and lower antigen clearance, which can be a problem for soluble antigens. Furthermore, surface expression of full-length Pfs25 on a single cell could allow the display of antigen for direct B cell recognition in close proximity to processed adenovirus antigen in the context of MHC to activate T cells, thus providing the requisite T cell help to enhance B cell activation. The high anti-yPfs25 antibody titers obtained 10 days post-immunization with Ad5-*pfs25* compared to titers following yPfs25/alum as well as the concurrent isolation of T-cells that produced IFNγ in response to restimulation with Ad5 is consistent with the dual activation of B and T cells. Previous work demonstrated that Ad vector-directed antigen expression induces significantly higher antibody titers than titers generated following co-immunization of an empty Ad vector and recombinant protein [36]. From this experiment the authors suggested that expression of a target antigen in the context of Ad5 transduction improves antibody titer and likely does so by providing T cell help in the lymph node follicle [36].

Next, to focus the immune response to specific Pfs25 epitopes Ad5-based vectors displaying Pfs25 epitopes within hypervariable regions (HVR) 1 or 5 of the hexon capsid protein on the viral vector surface (Ad5-HVRD2 or D3) were generated for use as booster immunizations following a prime with Ad5-*pfs25*. Other studies demonstrate that displaying antigen epitopes within these HVR domains of the viral capsid elicit antibodies to the target antigen [32, 37–39]. By expressing these Pfs25 epitopes within Hexon, which is the major viral capsid protein, 720 copies of the Pfs25 epitopes decorate the Ad5-HVR-pfs25 vector surface. Pfs25 epitopes (D2 and D3) from EGF domains DII and DIII, which have been implicated as the targets of transmission-blocking antibodies, were selected to display in Hexon HVRs 1 or 5 [16]. These viral vectors elicited high titer and high avidity anti-yPfs25 antibodies with similar IgG subclass profiles. However, the reduction in oocysts using serum obtained from mice boosted with Ad5-HVR5D2 was lower. The 12-amino acid D2 peptide was selected based on in silico homology with the Pvs25 structure and, perhaps, in Pfs25 is a slightly different epitope localizes to the loop of the EGF domain 2 [10, 40–42]. Further, a larger region of DII may need to be included in the D2 peptide since, in the original study implicating EGF domain II as a transmission-blocking target, domain II included a larger region of Pfs25 (amino acids 59–110) expressed in yeast [16]. By itself the recombinant Pfs25 domain II was poorly immunogenic, but a booster vaccination with a recombinant protein corresponding to the full exodomain of recombinant Pfs25 successfully induced transmission-reducing antibodies. It is possible that additional boosts with a larger region of Pfs25 would have increased the response to Ad5-HVR5D2. Indeed, serum obtained after boosting with recombinant yPfs25 in alum following a heterologous Ad5-*pfs25* priming immunization inhibited oocyst production as effectively as boosting with Ad5-HVR5D3. This finding is in marked contrast to the ineffectiveness of a homologous yPfs25/alum prime/boost immunization strategy, even though the same recombinant yPfs25 was used. These data strongly support the role of the Ad5 viral vector in enhancing the primary immune response and is the first demonstration of an Ad vector prime, recombinant protein boost strategy for Pfs25, although this has been suggested in the discussion of previous vaccine studies [43]. The success of this approach shows the promise of a strategy that combines the enhanced T cell activation of an Ad vector priming immunization with boosts using a recombinant protein vaccine [21, 44]. Future studies could include the evaluation of the longevity of the response and optimization of the viral vector and dosing schedule.

This study has highlighted the role for the D3 epitope as a transmission-blocking candidate since a booster immunization with Ad-HVR5D3, which displays only the 13 amino acid D3 peptide on the viral capsid, generates a transmission-reducing antibody response that is comparable to a booster immunization with the entire recombinant yPfs25 protein. The efficacy of this domain is consistent with previous findings demonstrating recognition of the D3 domain by two different Pfs25 specific transmission-blocking antibodies 4B7 and 1D2 [15–17]. However, the previous attempt to induce transmission-blocking antibodies using yeast-produced recombinant protein corresponding to the entire Pfs25 EGF domain III (amino acids 107–156) was not effective [16]. It is possible that limiting the domain to just the transmission-blocking epitope could serve to focus the response. Moreover, incorporating the peptide within the viral capsid allows for stronger signaling through interactions between the multiple Pfs25 epitopes displayed on the capsid and multiple BCRs, as well as taking advantage of the immuno stimulatory properties of the Ad vector. These Ad5-*pfs25* prime and Ad5-HVR capsid display vectors could also be used to screen additional Pfs25 peptides or a peptide library for transmission-blocking epitopes. Purified Ad5-HVR-Pfs25 peptide virions could be directly screened for recognition by transmission-blocking monoclonal antibodies or the ability to deplete transmission-blocking antibodies from serum. Additionally, Ad5-HVR-Pfs25 peptide antiserum could be tested for gamete surface recognition or transmission-blocking activity by SMFA. Taken together, these approaches could efficiently identify TBV epitopes that could be directly tested alone or in combination for the ability to induce malaria transmission-blocking antibodies.

## Conclusion

This study has identified several new Ad5-based Pfs25 vectors to enhance the humoral immune response to Pfs25. Compared to the recombinant yPfs25-alum vaccine, Ad5-*pfs25* elicited high Pfs25-specific IgG titers with greater transmission-blocking activity making Ad vectors a superior method for primary immunization. This observed improvement in the anti-Pfs25 response may be due to the Ad5-specific T cell response induced by Ad5-*pfs25* vaccination. This robust primary immune response mediated by Ad5-*pfs25* was exploited by combining it with boost vaccinations using Ad-based vectors that display relevant, transmission-blocking Pfs25 epitopes on its surface (Ad5-HVR5D3 or D2). The heterologous prime-boost approach increased the Pfs25-specific antibody titer, relative avidity, and transmission-reducing activity as compared to homologous prime-boost with Pfs25-alum. These novel transmission-blocking vaccine vectors provide approaches to augment and focus anti-Pfs25 immunity.

## Abbreviations

Ad5: adenovirus type 5
ChAd63: adenovirus isolated from chimpanzees
AD: 1× assay diluent
ConA: Concanavalin A
CMV: cytomegalovirus
DMEM: Dulbecco’s Modified Eagle Medium
ELISA: enzyme-linked immunosorbent assay
EGF: epidermal growth factor
FBS fetal bovine serum GPI: glyceryl-phosphatidylinositol
GFP: green fluorescent protein
HVR: hexon hypervariable regions
IFNγ: interferon gamma
IFA: immunofluorescence assay
Ig: immunoglobulin molecule
MVA: modified Vaccinia virus
mAb: monoclonal antibody
OD: optical density
D2: Pfs25 amino acids 83–95
D3: Pfs25 amino acids 122–134
PBS: phosphate buffered saline
SMFA: standard membrane-feeding assay
TO: tet operator
TBV: transmission-blocking vaccine
yPfs25: yeast-produced recombinant Pfs25
